# Detecting the Presence of Different Types of Oil in Seawater Using a Fluorometric Index

**DOI:** 10.3390/s19173774

**Published:** 2019-08-31

**Authors:** Emilia Baszanowska, Zbigniew Otremba

**Affiliations:** Department of Physics, Gdynia Maritime University, Gdynia 81-225, Poland

**Keywords:** oil pollution, oil detection, seawater, oil in seawater, excitation–emission spectra, oil fluorescence, fluorometric index

## Abstract

This study analyzed the fluorometric laboratory tests for the detection of the presence of oil in seawater in cases corresponding to the real situation in the sea: when the point of seawater sampling is not in the same place as the appropriate sensor. The phenomenon of fluorescence exhibited by both natural and alien substances (oil) in the sea was used. The possibility of oil detection in the water column based on a fluorometric index (FI) extracted from the excitation–emission matrix (EEM) was studied. Laboratory tests were carried out on water taken from the Gulf of Gdańsk (Baltic Sea). Seawater samples were contaminated with small amounts of various types of oil (the lowest oil-to-water ratio was 0.5 × 10^−6^). A statistically significant difference was found between FI values for uncontaminated seawater and seawater exposed to various kinds of oil (i.e., crude oils, lubricant oils, and fuels).

## 1. Introduction 

Oil pollution is an ongoing problem for the marine environment. However, this issue was significantly reduced as a result of the MARPOL Convention [1]: tanker construction was improved, strict standards for oil discharges were established, a legal system of deterioration was developed, and methods for the early detection of oils on the sea surface were improved. Instrumental analytical methods for detecting the perpetrators of spills were also regulated [2]. The technical and organizational basis for combating spillages has significantly improved [3,4]. However, oil spills still appear [5]. Currently, in the scientific literature, the issues of remote detection of oil stains appearing at sea are widely discussed [6,7,8]. Both optical and radar methods [3,9,10,11,12,13,14] are well developed in this field. Activities aimed at limiting the share of oil substances polluting the marine environment also include the possibility of early detection before they spread in the sea. This is particularly difficult if these substances exist in the depths of the sea in a dispersed form or they penetrate deeply into the environment beneath the surface of the water from tanks, pipelines, wrecks, mining equipment, or bottom seepage. In cases when the oil has spread in the sea and flows deeper into the sea under the influence of the wind and waves, different methods based on UV-visible, IR, and Raman spectroscopy, or those based on oil fluorescence [15,16,17,18], are required for the detection of oil substances. Moreover, for underwater oil pollution detection, different sensors can be used [19,20,21,22,23].

Both seawater and some hydrocarbon substances leaching into the water from the oil are capable of fluorescence induced by ultraviolet light. If seawater did not exhibit fluorescence, it would be easy to detect substances derived from oil. However, since oil substances and substances naturally occurring in the sea fluoresce similarly [18], a universal method for interpreting the fluorescence spectra in terms of detecting the presence of oil in seawater is required. Preliminary studies on seawater samples contaminated with only one type of oil (crude oil *Petrobaltic*), but sampled in various terms [24], have shown the possibility of using a numerical indicator extracted from the excitation–emission matrix (EEM) spectrum, whose value indicates the lack of exposure of water to oil pollution or an oil hazard in the vicinity of the point of water sampling or a possible sensor location. This paper outlines the results of a wider analysis of the possibility of signaling the presence of various types of oil in seawater, using a similar procedure for the interpretation of EEMs in all cases. The results presented in this paper are promising, but in order to finally implement the proposed method in real conditions and further in various sea regions, additional verification analyses are needed.

## 2. Materials and Methods

### 2.1. Materials

#### 2.1.1. Seawater Samples 

Seawater samples were collected in the Orłowo pier in Gdynia (Southern Baltic Sea, Poland). Seawater was sampled from a 1 m depth into 1 L glass bottles, three times in 2019: March (T = 4 °C and salinity 7.4 PSU), April (T = 6 °C and salinity 7.6 PSU), and May (T = 10°C and salinity 7.5 PSU). 

#### 2.1.2. Oil Samples

Several kinds of oils were used for the laboratory contamination of seawater. 

(1) Crude oils: 

Petrobaltic—extracted from the Baltic Sea shelf, light crude, with American Petroleum Institute API gravity 43–44° and sulfur content 0.12%. 

Flotta—extracted from the North Sea (Orkney), medium crude with API gravity 35.4° and sulfur content 1.22%.

Gullfaks—extracted from the North Sea (off-shore), light crude with API gravity 37.5° and sulfur content 0.22%.

(2) Lubricant oils:

Marinol 1240—as a representative of lubricant oil used for the lubrication of inverter-type marine engines operating on light fuel. The oil base contains less than 3% polycyclic aromatic hydrocarbons (PCA).

Cyliten 460—as a representative of lubricant oil used in marine ship engine systems and used for the lubrication of single- and multi-cylinder reciprocating compressors of synthesis gas. The oil base contains less than 3% PCA.

(3) Fuels:

Eurodiesel—used as fuel for diesel engines with a sulfur content of 10 mg/kg.

E95—light fuel with up to 1% benzene, <3% n-hexane, and approximately 6% toluene.

### 2.2. Methods

#### 2.2.1. Exposure of Seawater Samples to Oil

**Figure 1:**
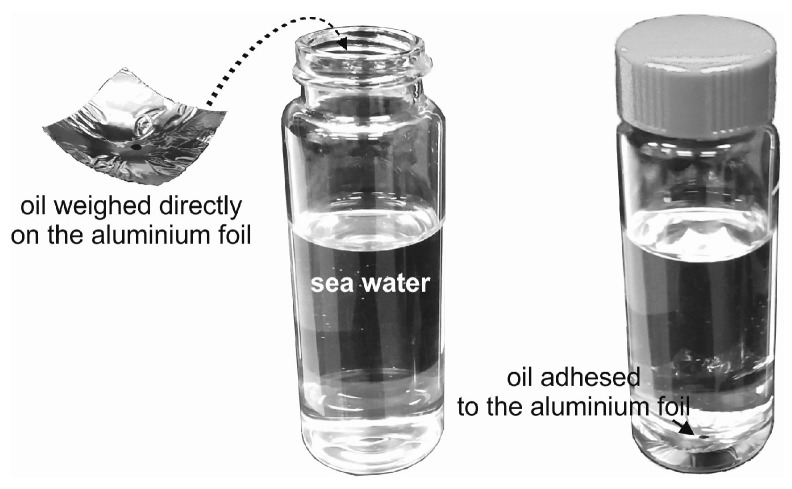
Procedure of the seawater sample exposure to oil.

For laboratory measurements, seawater samples were contaminated by each oil. Small amounts of oil were placed on a slice of aluminum foil and then weighed and inserted into a seawater sample (Figure 1) to reach the desired oil-to-water ratio. Four samples polluted by each kind of oil with an oil-to-water ratio (r_o/w_) in the range of 0.5 × 10^−6^ to 500 × 10^−6^ were prepared. Natural seawater was exposed to the added oil for one day.

#### 2.2.2. Measurement and Apparatus

##### Instrument 

A Hitachi F-7000 FL spectrofluorometer was used to determine the EEMs using a 1 × 1 cm quartz cuvette. 

##### Measurement Parameters 

The excitation wavelength was changed from 200 to 480 nm with an excitation sampling interval of 5 nm. The emission wavelength was changed from 260 to 600 nm with a 5 nm emission sampling interval, a 10 nm excitation slit, and a 10 nm emission slit. The integration time was 0.5 s, and the photomultiplier tube voltage was 400 V.

#### 2.2.3. Measurement of Seawater and Oil-Contaminated Seawater Samples

1. The EEMs for samples of environment seawater were previously measured three times, allowing for a reliable, artefact-free background to be obtained.
2. Next, the EEMs of seawater exposed to each kind of oil for various r_o/w_ were measured.
3. Measurements for all samples were performed at a stabilized temperature of 20 °C.
4. Rayleigh scattering to yield a digital matrix of EEMs was removed (if the excitation wavelength and emission wavelengths were equal and the emission wavelength was two times higher than the excitation wavelength).

## 3. Results and Discussion

### 3.1. Fluorometric Index (FI) Calculations

**Figure 2:**
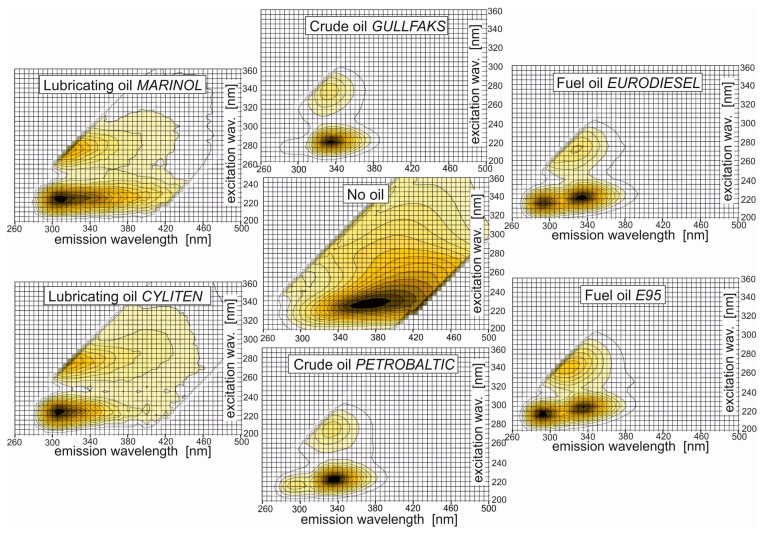
Excitation–emission matrix (EEM) spectra of seawater sampled on 15 March 2019, and the same seawater artificially polluted by individual oils for an oil-to-water ratio of 50 × 10^−6^. Each spectrum is normalized to its maximal peak.

The studies took into account 84 EEMs for artificially contaminated samples of seawater and nine EEMS for uncontaminated samples (Figure 2). Based on the EEMs of seawater samples and seawater contaminated by different kind of oils, an FI was proposed for oil detection. The proposed FI was extracted from the EEMs and used only specific values of fluorescence intensity described by the excitation wavelength maximum (λEx) corresponding to the emission wavelength maximum (λEm) for selected wavelengths for natural seawater and seawater polluted by oil. The FI was defined as a quotient of the fluorescence intensity at the emission wavelength for seawater polluted by oil to the intensity at the emission wavelength for natural seawater corresponding to the detected excitation maximum for both natural seawater and seawater polluted by oil described as FI_o/w_ by Formula (1). This definition of FI_o/w_ indicates the growth of its value in the presence of oil in water.

(1) $$FI_{o/w}={\left[\frac{I(\lambda_{Emission\;of\;seawater\;polluted\;by\;oil})}{I(\lambda_{Emission\;of\;natural\;seawater})}\right]}_{\lambda_{Excitation}}.$$

The effectiveness of oil detection in seawater based on the proposed indicator FI_o/w_ was checked, taking into account the kinds of oils (Section 2) and oil-to-water ratios.

For oil detection in seawater, it is necessary to take into account the presence of natural seawater components. Therefore, to obtain information on the presence of natural seawater components, the EEMs of natural seawater were determined three times in 2019. Next, the influence of oil presence in seawater on the EEMs was analyzed. Figure 2 presents EEMs for natural seawater (no oil) and seawater polluted by different types of oils selected for an oil-to-water ratio of 50 × 10^−6^ on 15 March 2019. The EEMs of natural seawater (no oil) in Figure 2 indicate the presence of the main peak in the UV-range centered at 225 nm of an excitation wavelength and corresponding to an emission wavelength centered at 360 nm (λ_Ex_/λ_Em_ = 225/360) (Table 1). The detected peak is well linked to the tryptophan-like seawater component. Figure 2 clearly presents the influence of oil on the EEMs. In the EEMs of seawater polluted by oil, for all oils, the main peak is centered at 340 nm for an emission wavelength corresponding to an excitation wavelength of 225 nm (λ_Ex_/λ_Em_ = 225/340) (Table 1) with an accuracy of ±5 nm. Moreover, for all types of oils, a second peak was centered at 330 nm for an emission wavelength corresponding to an excitation wavelength of 275 nm (λ_Ex_/λ_Em_ = 275/330) (Table 1) with an accuracy of ±5 nm, but with a lower fluorescence intensity than the main peak at 225/340. The results of the determined peaks for the EEMs for individual oils are presented in Table 1, and it is visible that, for the selected oil in the EEMs, other specific peaks depending on the type of oil and oil-to-water ratio are determined. The EEMs for all considered oils (Figure 2) revealed bands of fluorescence intensity between 300 and 400 nm, with the main band at 340 nm. These bands are probably caused by the presence of fluorophores, which are usually centered in one- to three-ring aromatic structures in the petroleum substances [25,26,27,28]. The EEMs from 15 March 2019 overlap with those from 15 April and 20 May 2019. 

Taking into account the determined EEM peaks for all oils, it is clear that the peak for natural seawater (λ_Ex_/λ_Em_ = 225/360) and the main peak for seawater polluted by oil (λ_Ex_/λ_Em_ = 225/340) were detected at the same excitation wavelength. However, taking into account the peaks for the EEMs determined previously for natural seawater from more than a dozen tests in 2017 [24], the peak for natural seawater is dominant for an emission wavelength at 355 nm (λ_Ex_/λ_Em_ = 225/355). This is a reason to choose the selected wavelengths for FI_o/w_ definition. Therefore, FI_o/w_ was calculated as the quotient of the fluorescence intensity at a 340 nm emission wavelength and an intensity at 355 nm, while the excitation wavelength remained equal to 225 nm (Formula (2)):

(2) $$FI_{o/w}={\left[\frac{I\left(\lambda_{Em=340}\right)}{I\left(\lambda_{Em=355}\right)}\right]}_{\lambda_{Ex=225}},$$

where I(λ_Em_) describes, respectively, the fluorescence intensity corresponding to the emission wavelength for polluted seawater (340 nm) and natural seawater (355 nm) linked to the same excitation wavelength (λ_Ex_) for both kinds of seawater (225 nm). 

Calculations for the FI_o/w_ values were performed for both natural seawater samples and seawater samples polluted by individual oils (one time for each sample for three different sampling times). Table 2, Table 3, Table 4 and Table 5 present the calculated FI_o/w_ values for three different times in 2019. The results for the calculated FI_o/w_ values indicate the difference between natural seawater and polluted seawater. FI_o/w_ for polluted seawater achieved higher values (above 1) than for natural seawater (below 1). This is confirmation of the good choice of wavelengths for FI_o/w_. Moreover, in Table 3, Table 4 and Table 5, it is clearly visible that FI_o/w_ values depend on the type of oil and are stabilized for an oil-to-water ratio above 50 × 10^−6^. 

**Figure 3:**
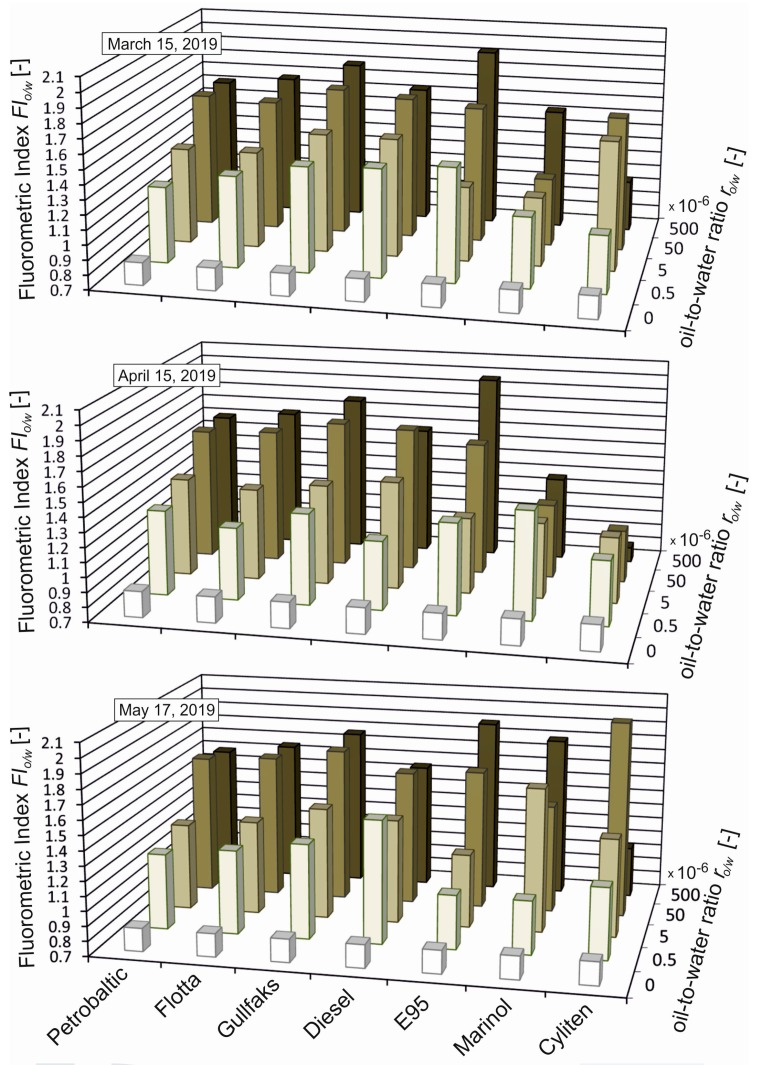
Fluorometric index (*FI_o/w_*) for unpolluted seawater and seawater artificially polluted with various kinds of oil for oil-to-water ratios of 0.5 × 10^−6^, 5 × 10^−6^, 50 × 10^−6^, and 500 × 10^−6^.

Figure 3 presents *FI*_o/w_ for unpolluted seawater and seawater polluted with various kinds of oil for different oil-to-water ratios ranging from 0.5 × 10^−6^ to 500 × 10^−6^. A distinct increase in *FI*_o/w_ already exists for r_o/w_ = 0.5 × 10^−6^. Beginning with r_o/w_ = 50 × 10^−6^, the *FI*_o/w_ value ceases to depend on r_o/w_. The deviation of *FI*_o/w_ values from other oils was observed for Marinol and Cyliten. This is probably caused by the low content of PCA.

### 3.2. Statistical Analysis of the Fluorometric Index 

**Figure 4:**
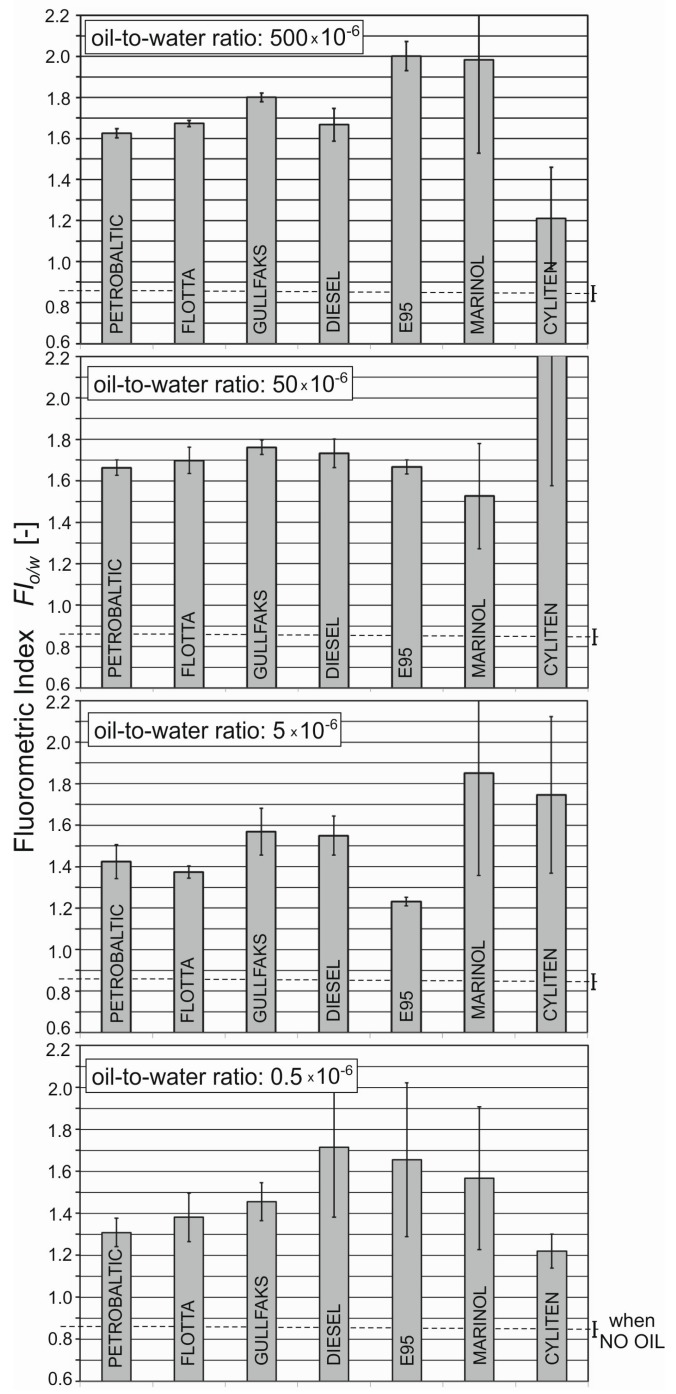
Mean values of fluorometric index confidence intervals of the mean (the significance level was 0.05) for natural seawater (no oil) and seawater artificially polluted by different kinds of oil for oil-to-water ratios of 0.5 × 10^−6^, 5 × 10^−6^, 50 × 10^−6^, and 500 × 10^−6^.

Statistical calculations were performed to check the deviation of determined fluorometric index (*FI*_o/w_) values for three different times of sampling for natural seawater and the same seawater polluted by oil. Confidence intervals (CI) of the mean of the *FI*_o/w_ values were performed using a Student’s *t*-test at a significance level of 0.05 for each kind of oil for three water sampling terms. Figure 4 presents the fluorometric index (*FI*_o/w_) and CI of the mean for natural seawater (no oil) and seawater artificially polluted by different kinds of oil with oil-to-water ratios of 0.5 × 10^−6^, 5 × 10^−6^, 50 × 10^−6^, and 500 × 10^−6^. The *FI*_o/w_ values for different kinds of oils and different oil-to-water ratios are statistically different in several cases. However, the confidence bars of contaminated water and clean water did not overlap. 

**Figure 5:**
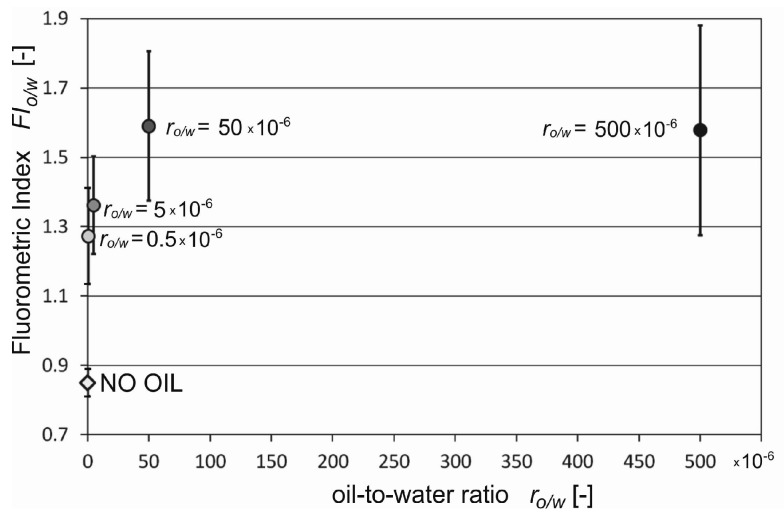
Fluorometric index (*FI_o/w_*) with standard deviation of the mean for natural seawater (no oil) and seawater artificially polluted by various kinds of oil.

Next, the standard deviation of the mean values of *FI*_o/w_ for all kinds of oil for 21 samples from three different times of seawater sampling were determined. Figure 5 presents the average standard deviation of the mean calculated *FI*_o/w_ values for natural seawater and artificially polluted seawater for different oil-to-water ratios. The highest value of the standard deviation of *FI*_o/w_ value was found for the 500 × 10^−6^ oil-to-water ratio. However, the standard deviation of the average *FI*_o/w_ value for water exposed to oil does not overlap with the standard deviation of the average *FI*_o/w_ value for uncontaminated water. Moreover, the coefficient of variations of *FI*_o/w_ achieved values of approximately 10% for the oil-to-water ratios 0.5 × 10^−6^ and 50 × 10^−6^, 14% for the oil-to-water ratio 50 × 10^−6^, and 19% for the oil-to-water ratio 500 × 10^−6^. This indicates that the diversity of *FI*_o/w_ for all oil samples within a given oil-to-water ratio is small, and the *FI*_o/w_ values indicate high similarity.

## 4. Conclusions

The value of the fluorometric index (*FI*_o/w_) indicates the presence of oil in seawater, although it nonlinearly depends on the oil-to-water ratio, but only for low values. The *FI*_o/w_ values remain at the same level when the oil-to-water ratio exceeds 50 × 10^−6^. The influence of the type of oil does not manifest itself in an unambiguous way. However, the performed statistical calculations indicate a high similarity in the *FI*_o/w_ values independent of the type of oil as well as the oil-to-water ratio. Moreover, no case was found where the value of *FI*_o/w_ for seawater exposed to oil was not different from the value of *FI*_o/w_ for natural seawater. At an oil-to-water ratio below 0.5 × 10^−6^, the detection of oil with the *FI*_o/w_ indicator is probably possible; however, the quantitative lower limit of detection is not yet specified. In turn, to check the effectiveness of the method against weathered oils and their mixtures, separate research is needed. A possible future-oriented immersion sensor in its structure would have to reflect the idea described in this work (i.e., be equipped with an excitation channel (225 nm) and two emission channels (340 and 355 nm)). The listed wavelengths refer to the area of the Gulf of Gdańsk (Baltic Sea); therefore, in relation to other sea areas, the values of these wavelengths should be verified and possibly corrected.

## Figures and Tables

**Table 1:** Major fluorescent peaks of natural seawater and the same samples polluted by oil at oil-to-water ratios of 0.5 × 10^−6^, 5 × 10^−6^, 50 × 10^−6^, and 500 × 10^−6^ with their wavelength-independent maxima (for 15 March 2019).

Ex_max_ (nm) ± 5 (nm)/Em_max_ (nm) ± 5 (nm)				
Petrobaltic	Peak 1	Peak 2	Peak 3	Peak 4
0.5 × 10^−6^	225/330	220/310	255/360	270/320
5 × 10^−6^	225/340		255/360	270/325
50 × 10^−6^	225/340	220/295		275/335
500 × 10^−6^	225/340	220/295		275/335
Flotta				
0.5 × 10^−6^	225/340		250/360	275/325
5 × 10^−6^	225/340		250/360	275/330
50 × 10^−6^	220/335			275/335
500 × 10^−6^	220/335			275/335
Gullfaks				
0.5 × 10^−6^	225/335		255/360	275/320
5 × 10^−6^	225/340			275/330
50 × 10^−6^	220/335			275/335
500×10^−6^	220/335			275/335
Eurodiesel				
0.5 × 10^−6^	225/335	225/310		275/320
5 × 10^−6^	225/340	225/310		275/335
50 × 10^−6^	220/335	220/295		275/330
500 × 10^−6^	220/335	215/290		275/335
E95				
0.5 × 10^−6^	225/335	220/310		275/325
5 × 10^−6^	225/335	225/310		275/325
50 × 10^−6^	225/335	220/295		270/320
500 × 10^−6^		215/290		260/310
Marinol				
0.5 × 10^−6^	225/335			275/320
5 × 10^−6^	225/335			275/320
50 × 10^−6^	225/340	225/310		275/320
500 × 10^−6^		225/305		275/320
Cyliten				
0.5 × 10^−6^	225/340	225/305		275/320
5 × 10^−6^		225/305		275/320
50 × 10^−6^		225/305		275/320
500 × 10^−6^	225/340	225/305		275/320

**Table 2:** Fluorometric index (*FI_o/w_*) calculated by Formula (2) for the uncontaminated seawater.

FI_w/o_ (-)		
15 March 2019	15 April 2019	20 May 2019
0.85	0.88	0.85

**Table 3:** Fluorometric index (*FI_o/w_*) calculated by Formula (2) for the seawater contaminated with individual oils on 15 March 2019.

r_o/w_	*FI*_o/w_ (-)						
	Petrobaltic	Flotta	Gullfaks	Eurodiesel	E95	Marinol 1240	Cyliten N-460
0.5 × 10^−6^	1.22	1.33	1.42	1.44	1.48	1.18	1.09
5 × 10^−6^	1.36	1.36	1.52	1.51	1.21	1.17	1.58
50 × 10^−6^	1.63	1.60	1.72	1.68	1.64	1.17	1.62
500 × 10^−6^	1.62	1.67	1.79	1.64	1.93	1.53	1.05

**Table 4:** Fluorometric index (*FI_o/w_*) calculated by Formula (2) for the seawater contaminated with individual oils on 15 April 2019.

r_o/w_	*FI*_o/w_ (-)						
	Petrobaltic	Flotta	Gullfaks	Eurodiesel	E95	Marinol 1240	Cyliten N-460
0.5 × 10^−6^	1.28	1.20	1.33	1.17	1.33	1.44	1.14
5 × 10^−6^	1.38	1.33	1.39	1.44	1.22	1.22	1.15
50 × 10^−6^	1.60	1.62	1.71	1.69	1.61	1.21	1.06
500 × 10^−6^	1.60	1.65	1.77	1.58	1.97	1.27	0.8

**Table 5:** Fluorometric index (*FI_o/w_*) calculated by Formula (2) for the seawater contaminated with individual oils on 20 May 2019.

r_o/w_	*FI*_o/w_ (-)						
	Petrobaltic	Flotta	Gullfaks	Eurodiesel	E95	Marinol 1240	Cyliten N-460
0.5 × 10^−6^	1.21	1.27	1.34	1.54	1.07	1.06	1.18
5 × 10^−6^	1.29	1.34	1.46	1.41	1.20	1.68	1.37
50 × 10^−6^	1.65	1.67	1.75	1.62	1.65	1.44	2.04
500 × 10^−6^	1.59	1.65	1.77	1.55	1.89	1.79	1.04
